# Study on Life Satisfaction of the Elderly Based on Healthy Aging

**DOI:** 10.1155/2022/8343452

**Published:** 2022-10-03

**Authors:** Hua Tian, Jie Chen

**Affiliations:** ^1^College of Life Science, Xinyang Normal University, Xinyang, China; ^2^School of Marxism, Xinyang Normal University, Xinyang, China

## Abstract

The life satisfaction of the elderly is the key to subjective well-being and healthy aging. Many related studies are focused on the affected factors, including health status, economic level, social support, pension mode, social security, and intergenerational support, etc., but few are based on the macro perspective of healthy aging. This study constructed a healthy aging evaluation system with 6 dimensions, including 15 primary indicators and 57 secondary indicators, to evaluate the relationship between healthy aging and elderly life satisfaction. Results showed that the 13168 participants were, mainly, female (53.76%), 80–99 years old (47.99%), lived in rural areas (77.00%), married and living with their spouse (43.70%), and widowed (52.15%). 80.32% lived with household members. 70.37% elderly were satisfied with their lives. Specifically, there was no gender difference in life satisfaction of the elderly (*p*=0.273), but there were significant differences between groups of urban and rural (*p* < 0.001), age groups of 65–79 and 80 older (*p* < 0.001), marriage groups of unmarried and married (*p* < 0.001), and types of elderly care of living alone and with others (*p* < 0.001), respectively. Among the six dimensions of healthy aging, healthcare performed best and living environment dimension was the worst, which was an area that urgently needed to strengthen. The odds ratios (ORs) showed that the dimensions of social participation/social equity and economic finance played important roles in the well-being of the elderly. Under the macro background of healthy aging, how to take measures from the micro perspective of the healthy aging evaluation index system and ultimately improve the life satisfaction of the elderly and still needs to be explored in-depth.

## 1. Introduction

To cope with global aging, the World Health Organization (WHO) has proposed a Decade of Healthy Aging 2020–2030 to promote the health of more than a billion people aged 60 and over [1]. The Chinese government has learned from and adopted the concept of healthy aging from the WHO, which defines healthy ageing as “developing and maintaining the functional ability to make older people well-being”. WHO stressed the need to create a world caring for the elderly, and the health system changed from a disease-based medical model to a comprehensive care model focusing on the needs of the elderly, especially, transportation, housing, social protection and support, urban development, information and communication, education, labor health and long-term care, and other multisectoral collaborative promotion [2].

The aging process is a stage of the life cycle. In some cases, there are significant differences between individuals in the physical and cognitive state of the elderly [3], which leads to the loss of cognitive and physical abilities. Life satisfaction and physical activity are two key aspects of healthy aging [4]. Life satisfaction of the elderly is the concrete embodiment of subjective well-being and healthy aging [5], and the theoretical basis for the development of healthy aging and the implementation of the healthy China strategy in China. Some studies have been used to explain the life satisfaction of the elderly [6]. The main factors that affect the life satisfaction of the elderly are health status, economic level, social support, pension mode, social security, and intergenerational support, etc. One study has shown that life satisfaction and happiness increase with age [7]. The life satisfaction of the elderly living with their family is significantly higher than that of those living alone. Individuals with superior economic conditions and maintaining physical activity and social relations show higher life satisfaction than others [8]. The more chronic diseases the elderly have, the lower their life satisfaction [9]. Although a large number of studies have focused on the life satisfaction of the elderly, there are little studies on how to scientifically and reasonably quantify the healthy aging index and then carry out the life satisfaction evaluation of the elderly based on healthy aging. Based on previous research [10], six dimensions of medical care, health education, living environment, road transportation, social participation and social equity, economy and finance, including 15 primary indicators and 57 secondary indicators, were constructed to objectively evaluate the correlation of healthy aging with life satisfaction of the elderly, which covers 13168 elderly aged 65–117 in 23 provinces and municipalities directly under the Central Government in China, including 6089 males and 7079 females.

## 2. Methods

### 2.1. Participants

The participants were drawn from the Chinese Longitudinal Healthy Longevity Survey (CLHLS-2018), aged 65 and above. CLHLS randomly selected samples from 23 representative provincial cities and autonomous regions in China. After deleting the samples with missing and invalid variable values, 13168 effective samples were selected. CLHLS-2018, carried out by the China Center for Disease Control and Prevention and the Research Center for healthy aging and development of the National Development Research Institute of Peking University, aimed to determine the health status and factors of the elderly in many aspects, so as to find potential health problems.

### 2.2. Measures

The fully aligned polygonal graphical indexing method was used to deal with the situation of healthy aging in different provinces. The fundamental principle of the method is that indices are set up and the higher limit value of these indices is taken as the radius to construct a central inside shape [11]. See the literature for the specific calculation process [12].

### 2.3. Variables

The process of healthy aging in China includes 6 dimensions, 15 primary indices and 57 secondary indices (Table 1). The data are from China Statistical Yearbook, China Urban Construction Statistical Yearbook and China Health Statistical Yearbook. If *S* < 0.5, 6 dimensions and 15 primary indices are defined as 0, or is defined as 1. The life satisfaction and health data of the elderly are from the Chinese Longitudinal Healthy Longevity Survey (CLHLS-2018), based on the questions of “What do you think of your life now”. This question has six answers of “very good”, “good”, “general”, “poor”, “very poor” and “I do not know”. When screening the samples, the participants who answered “I do not know” were eliminated. Thus, there were only five answers. When calculating the binary regression equation, “very good” and “good” were merged to be defined as 1. The other three were defined as 0.

## 3. Results

### 3.1. Participants Characteristics

Of the 13168 participants (Table 2), 7079 (53.76%) were female, and 6089 (46.24%) were male. The proportion of males and females was almost equal. The main age groups were 65–79 (39.57%) and 80–99 (47.99%). The vast majority (77.00%) lived in rural areas. The marital statuses of the elderly were mainly married and living with a spouse (43.70%) and widowed (52.15%). 10576 (80.32%) lived with household members. The samples were widely distributed in 23 provinces cities and autonomous regions. The four main provinces were Shandong (1668), Guangxi (1603), Jiangsu (1575), and Sichuan (1135).

### 3.2. Relationship between Participant Characteristics and Life Satisfaction

Table 3 was the relationship between participant characteristics and life satisfaction. Excepting living alone, participants based on demographic variables of residence, age, marriage, and living with others had all higher life satisfaction, accounting for more than 60%. There were significant differences between groups of urban and rural (*p* < 0.001), age groups of 65–79 and 80 older (*p* < 0.001), marriage groups of unmarried and married (*p* < 0.001), and types of elderly care of living alone and with others (*p* < 0.001) respectively. However, there was no difference between males and females (*p*=0.273). Overall, 9266 (70.37%) participants thought life was satisfactory, dissatisfaction with life accounted for only 29.63%. There was a significant difference between the satisfied group and the dissatisfied group (*p* < 0.001).

### 3.3. Life Satisfaction of the Elderly Based on Healthy Aging

Among the six dimensions of healthy aging (Table 4), the dimension of healthcare performed best (*S* ≥ 0.5) and the dimensions of the living environment, road traffic, and social participation/social equity were all worse, calculated using the fully aligned polygonal graphical indexing method. For the elderly numbers of life satisfaction under the condition of *S* ≥ 0.5, the participant numbers who were satisfied with life was the highest in the dimension of healthcare, up to 52.5%. Next is health education, and the living environment dimension has the least number of elder people who were satisfied with life, only is 541(4.11%). Thus, the dimension of the living environment performed worst and needed to be improved to improve the elderly's satisfaction with their lives. Under the background of healthy aging, how to take measures and ultimately improve the life satisfaction of the elderly still needs to be explored in-depth.

### 3.4. Relationship between Life Satisfaction and Six Dimensions of the Elderly

This work compared and analyzed six dimensions affecting the life satisfaction of the elderly, such as healthcare, health education, road traffic, social participation/social equity, and economic finance (Table 5). For healthcare, in the life satisfaction group, 6913/9266 (74.61%) were satisfied with healthcare, while in the life dissatisfied group, 2417/3902 (61.94%) were satisfied with healthcare. Compared to the life satisfaction and dissatisfaction groups, the possibility of life dissatisfaction brought by healthcare is far less likely to bring life dissatisfaction [OR = 0.535(0.480–0.598, *p* < 0.001)]. The adjusted OR for the satisfied group is 0.565(0.505–0.632, *p* < 0.001). For the other five dimensions, the adjusted OR were, respectively [OR = 0.813(0.725–0.913, *p* < 0.001)], [OR = 0.247(0.181–0.338, *p* < 0.001)], [OR = 0.212(0.169–0.267, *p* < 0.205)], [OR = 0.871(0.739–1.027, *p* < 0.001)], [OR = 3.823(3.039–4.809, *p* < 0.001)]. For the dimension of social participation/social equity, 1108/9266 (11.96%) and 497/3902 (12.74%) participants were in the satisfied and dissatisfied groups, respectively. For the dimension of economic finance, the two figures were 2482/9266 (26.79%) and 1001/3902 (25.65%). Adjusted for gender, residence, age, nationality, and living status, the ORs of healthcare, social participation/social equity, and economic finance have all gone up. This also showed that the dimensions of healthcare, social participation/social equity, and economic finance play a very important role in the well-being of the elderly, especially the last two.

## 4. Discussion

Measuring and monitoring healthy aging in countries can promote a common understanding of the types of effective or ineffective initiatives to promote healthy aging [13]. Medical treatment, economy, and finance are important areas to improve the development level of healthy aging. Also, health education, living environment, road transportation, social participation/social equity cannot be ignored. In fact, these indices will comprehensively affect the development level of healthy aging in different regions and the life satisfaction of different elderly groups [14]. Healthcare expenditure is increasingly concentrated on the elderly group [15]. Especially in the period before death, the medical care expenditure increased sharply [16]. Medical insurance can significantly improve the health status and life satisfaction of the elderly [17]. Health education helps individuals to change unhealthy behavior and lifestyles, so as to reduce or eliminate the risk factors affecting health [18]. Health literacy is negatively correlated with medical utilization and expenditure. Public health strategies promoting appropriate education among patients with low health literacy (LHL) may help to improve health outcomes and reduce unnecessary medical visits and costs [19]. Collective health education activities are helpful to the health recovery of the elderly with complications [20], but the number of comorbidities may interfere with the effect of education and exercise programs [21]. When carrying out healthcare services, we should try to carry out appropriate training for the elderly in order to improve health literacy, cognitive function, and life satisfaction [22].

In addition to good healthcare and education, the living environment is also an important index to measure the urban per capita environmental quality, including the health, comfort, habitability, and happiness of the human settlement environment [23]. For example, an international study using data from 14 cities around the world found that building environmental attributes, including higher living density, good street network, availability of public transport, and more parks, are positively related to the health status of adults [24]. Urban green space improves the environmental quality of urban areas by regulating microclimates, reducing heat stress and atmospheric carbon dioxide, and enhancing biodiversity to improve the physical and mental health of urban residents and the elderly [25]. Furthermore, it is also an important development direction and goal to actively promote healthy aging and build a healthy aging-oriented urban human settlement environment design and assessment system [26].

After retirement, the elderly are likely to replace work travel with nonwork travel, and use more public transport. Relevant data, such as traffic travel need to be added, but they are not found. There is increasing evidence that walking can prevent various health problems, so as to help the elderly maintain their physical, cognitive independence and life satisfaction [27]. Transportation for the elderly is also an indispensable part of the concept of healthy aging [28]. Therefore, aging and transportation mode, aging and traffic safety, aging society, and intelligent transportation are facing challenges in highlighting the livability and humanity of the city, especially in adapting to the retirement life of the elderly. We also need to continuously improve the level of public services through institutional arrangements and give the elderly more opportunities and platforms for fair participation in social development and governance to ensure that the elderly have assessed to due social rights and services. Social participation is a key factor affecting the quality of life of the elderly, which is also a positive response to the construction of an aging environment to enhance the elderly's sense of participation, acquisition, and happiness [29]. Research shows that community environment and social participation have significant positive effects on the health of the elderly. At the same time, social participation plays an intermediary role in the relationship between the interpersonal environment and elderly health [30]. Economic finance is an important indicator of the security dimension of healthy aging. The direct pressure brought by the severe population aging situation is the life care of the elderly, which must be solved by the coordinated development of public financial support, pension supplement, and aging financial development of the government [31]. Therefore, the problem of aging is essentially an economic and financial problem, which is a necessary condition for the realization of healthy aging to a certain extent.

The limitations should not be ignored. First, due to the limitation of the CLHLS-2018 questionnaire, the life satisfaction of the elderly was evaluated by the one-dimensional question, without the life satisfaction scale. Second, the development index system of healthy aging includes multiple dimensions (see Table 6), and this manuscript only selected six key dimensions.

## 5. Conclusions

Our findings demonstrate that healthcare contributes the most to the life satisfaction of the elderly among six dimensions of healthy aging, while the other five aspects are relatively poor and need to be improved, especially the living environment dimension. Healthcare, social participation/social equity, and economic finance play important roles in the life satisfaction of the elderly.

## Figures and Tables

**Table 1:** Assessment index system of development level of healthy aging in China.

Dimension	Primary indices	Secondary indices	Unit
*Health care*	Medical expenditure	1. Per capita health expenditure	Yuan
		2. Proportion of medical and health expenditure in GDP	%
		3. Proportion of per capita healthcare expenditure in per capita consumption expenditure	%
		4. Urban and rural medical insurance participation rate	%
		5. Resident hospitalization rate	%
	Medical facilities	6. Hospitals per 10000 people	a
		7. Numbers of hospital beds per 1000 people	a
		8. The rate of utilization of hospital beds	%
		9. Numbers of beds in elderly care institutions per 1000 people	a
	Medical service	10. Numbers of doctors per thousand people	a
		11. Registered nurses per 1000 people	a
		12. Dependency ratio of elderly population	%
		13. Proportion of health examination in the total population.	%
		14. Proportion of the elderly in the number of health examination (Age ≥ 65)	%

*Health education*	Health education service	15. Health consultation and policy suggestions	a
		16. Public health education activities	a
		17. Health column jointly with the media	a
		18. Health information broadcast by media	a
	Health education materials	19. Plane health material production	a
		20. Audio visual health materials	×1000
		21. Health mobile short message	×1000
		22. Health material	×1000
	Health training	23. Sponsored health website	a
		24. Health training	a

*Living environment*	Environmental greening	25. Per capita urban green space area	Hectare
		26. Parks per 10000 people	a
		27. Per capita park green space area	m^2^
		28. Greening coverage rate of built-up area	%
		29. Urban population density	Person/square kilometer
	Resource processing	30. Harmless treatment rate of domestic waste	%
		31. Per capita emission of waste gas pollutants	Kg/person
		32. Per capita water resources	m^3^/person
		33. Per capita wastewater discharge	Ton/person
		34. Sewage treatment rate	%

*Road traffic*	Road	35. Urban road area per capita	m^2^
		36. Urban road length per 10000 people	Kilometer
	Transport	37. Number of taxis per 10000 people	A
		38. Public transport vehicles per 10000 people	A
		39. Per capita transportation expenditure	Yuan

*Social participation and social equity*	Public resource	40. Proportion of employed population in tertiary industry in total employed population	%
		41. Number of health, social security and social welfare employees per 10000 people	a
		42. Public library collections per capita	a
		43. Construction area of public library per 10000 people	m^2^
	Social conditions	44. Art performance venues per 10000 people	a
		45. Number of college students per 100000	a
		46. Consumer price index	Unit
		47. Proportion of internet broadband assess users in the total population	%

*Economic finance*	Economic income	48. Per capita local public budget income	Ten thousand yuan
		49. Per capita disposable income	Yuan
	Economic expenditure	50. Per capita local public budget expenditure	Ten thousand yuan
		51. Per capita consumption expenditure	Yuan
		52. Per capita public security expenditure	Yuan
		53. Per capita housing security expenditure	Yuan
		54. Per capita expenditure on culture, sports and media	Yuan
		55. Per capita expenditure on education	Yuan
	Commercial insurance	56. Commercial insurance depth	%
		57. Commercial insurance density	Ten thousand yuan/person

**Table 2:** Descriptive statistics and variables (*N* = 13168).

Characteristics		Frequencies (%)	Variables		Definition
*Sex*	Male	6089(46.24)	*Sex*	Male	1
	Female	7079(53.76)		Female	0

*Age*	65–79	5211(39.57)	*Age*	65–79	0
	80–99	6319(47.99)		>80	1
	>100	1638(12.44)	*Residence*	Urban	1
*Residence*	Urban	3028(23.00)		Rural	0
	Rural	10140(77.00)	*Marriage*	Unmarried	0
*Marriage*	Unmarried	119(0.90)		Married	1
	Married and living with spouse	5755(43.70)	*Types of elderly care*	Alone	0
	Married and not living with spouse	379(2.88)		With others	1
	Divorce	48(0.36)	*Life satisfaction*	Satisfied	1
	Widowed	6867(52.15)		Dissatisfied	0

*Types of elderly care*	With household member(s) alone	10576(80.32) 2158(16.39)	*6 dimensions*	*S* < 0.5	0
				*S* ≥ 0.5	1
	In an institution	434(3.30)			

*Note*. About life satisfaction, “satisfied” means the answer from the participant is “very good” or “good”. And “dissatisfied” means the answer from the participant is “general” “poor” or “very poor”.

**Table 3:** Participant characteristics (*N* = 13168).

Characteristic (total)	Numbers of elderly satisfied with life (%^*∗*^)	*P* Value
*Gender*		
Male (6089)	4256 (69.90)	0.273
Female (7079)	5010 (70.77)	

*Residence*		
Urban (3028)	2241 (74.01)	<0.001
Rural (10140)	7025 (69.28)	

*Age*		
65–79 (5638)	3864 (68.53)	<0.001
≥80 (7530)	5402 (71.74)	

*Marriage*		<0.001
Unmarried (2157)	1318 (61.10)	
Married (11011)	7948 (72.18)	

*Types of elderly care*		
Alone (119)	58 (48.74)	<0.001
With others(13049)	9208 (70.56)	

*Group*		
Satisfaction group	9266 (70.37)	<0.001
Dissatisfaction group	3902 (29.63)	

*Note*. ^*∗*^ Percentage refers to the ratio of numbers of elderly satisfied with life to the total number of people in this group.

**Table 4:** Life satisfaction of the elder based on healthy aging (*N* = 13168).

Items	*S* (mean ± SD)	Numbers^a^
Healthcare	0.582 ± 0.135	6913 (52.50%)
Health education	0.457 ± 0.181	3215 (24.42%)
Living environment	0.391 ± 0.050	541 (4.11%)
Road traffic	0.389 ± 0.117	1722 (13.08%)
Social participation/Social equity	0.390 ± 0.082	1108 (8.41%)
Economic finance	0.405 ± 0.129	2482 (18.85%)

*Note*. Numbers^a^ refers to the elderly numbers of life satisfaction under the condition of *S* ≥ 0.5.

**Table 5:** Six dimensions of life satisfaction (*N* = 13168). OR values are for the satisfied group (*n* = 9266) relative to dissatisfied group (*n* = 3902).

Six dimensions	Unadjusted OR^a^ (95% CI)	*P* Value	Adjusted OR^b^ (95% CI)	*P* Value
Healthcare	0.535 (0.480–0.598)	<0.001	0.565 (0.505–0.632)	<0.001
Health education	0.860 (0.768–0.963)	<0.001	0.813 (0.725–0.913)	<0.001
Living environment	0.253 (0.186–0.346)	<0.001	0.247 (0.181–0.338)	<0.001
Road traffic	0.244 (0.196–0.303)	<0.001	0.212 (0.169–0.267)	<0.001
Social participation/Social equity	0.808 (0.687–0.951)	<0.001	0.871 (0.739–1.027)	<0.001
Economic finance	3.288 (2.587–4.027)	<0.001	3.823 (3.039–4.809)	<0.001

Note: ^a^OR: odds ratio. ^b^Adjusted: adjusted for gender, residence, age, nationality, and living status.

**Table 6:** International influential representative health aging assessment index system.

Index system	Time	Issuing agency	Dimension
*Global age-friendly cities: a guide*	2007	World Health Organization	Architecture and environment, transportation, housing, social participation, respect and social inclusion, public participation and employment, communication and information, community support, and health services
*Age-friendly community*	2009	NYC department for the aging	Social environment, built environment, community and public participation, services, and facilities
*Well-being*	2009	Organization for economic cooperation and development	Material conditions, quality of life, and sustainability
*Active ageing index*	2012	United Nations Economic Commission for Europe	Work, social participation, independent, healthy and safe life, and the ability to build a positive aging environment
*Assessment system of healthy aging in large and medium-sized cities in China(2019–2020))*	2020	International Academy of Aging Sciences	Healthcare, living environment, transportation, social equity, economy, and finance
*National healthy city assessment index system*	2018	National Patriotic Health Campaign Committee	Healthy environment, healthy society, health services, healthy people, and healthy culture
*Old friendly city New York plan*	2008	New York Academy of Medical Sciences	Community and public participation, housing, public space and transportation, health and social services
*Healthy aging best city index*	2017	Milken Institute	Environmental livability, healthcare, financial security, education, convenient transportation, employment, living arrangement, and community participation
*Index system of elderly community assessment guide*	2015	Canadian public health agency	Outdoor space and buildings, transportation, housing, social participation, respect and social inclusion, citizen participation and employment, communication and information, community support and health services, elderly health, and social outcomes
*Age-friendly city*	2009	Manchester City Council	Housing, transportation, environment, community safety, employment and income, culture and learning, healthy aging, care and support services

## Data Availability

The research data used to support the findings of this study are available from the corresponding author upon request.
